# Perceptions of learning and teaching human movement in physiotherapy: A systematic review and metasynthesis of qualitative studies

**DOI:** 10.1080/17482631.2023.2225943

**Published:** 2023-06-26

**Authors:** Sirpa Ahola, Arja Piirainen, Pirjo Vuoskoski

**Affiliations:** a Faculty of Sport and Health Sciences, University of Jyväskylä, Jyvaskyla, Finland; b Physiotherapy, Metropolia University of Applied Sciences, Helsinki, Finland

**Keywords:** Metasynthesis, Qualitative, Physiotherapy, Learning and teaching, Human movement

## Abstract

**Purpose:**

Human movement is essential for health and well-being. Understanding human movement is pivotal in physiotherapy, but also an important element of physiotherapy education. This review identified, critically appraised, and synthesized the available evidence on learning and teaching human movement in physiotherapy as perceived by students, therapists, and instructors.

**Methods:**

The databases MEDLINE, CINAHL, ERIC, PsycINFO, MEDIC and FINNA, were searched. The search was conducted in March/April 2020 and updated in March 2022. The systematic review followed the JBI methodology for systematic reviews of qualitative evidence and was conducted in accordance with an a priori protocol.

**Results:**

The overall quality of the 17 included studies was scored low on ConQual but dependability and credibility were rated as moderate. Four synthesized findings aggregated from 17 categories and 147 findings described the perceived significance of 1) being present in movement, 2) movement quality, 3) movement transfer, and 4) contextual factors for the learning or teaching of human movement in physiotherapy.

**Conclusion:**

The synthesized findings indicate that the perceived significance of contextual factors, movement quality and transfer, and being present in movement should be considered in all learning and teaching of movement in physiotherapy. However, the evidence of the review findings was evaluated as low-level, which should be considered when applying these results to physiotherapy education or practice.

## Introduction

Physiotherapy in the 21^st^ -century is an emerging branch of health systems that are complex and constantly changing (World Physiotherapy, 2021). The foundations of physiotherapy education that aims to prepare graduates for contemporary practice, are also continuously evolving (ERWCPT, 2020). As highlighted by World Physiotherapy (2021), professional practice education under the supervision of appropriately qualified instructors, is an essential element of physiotherapist entry-level education programs, as it enables students to work with clients, groups, and communities in authentic environments (Delany & Bragge, 2009; Ernstzen et al., 2009; Lewthwaite et al., 2022; Stoikov et al., 2022). Whether the stage be education or professional practice, one of the constant phenomena encountered in physiotherapy is the learning and teaching of human movement, which is considered an essential element of health and well-being (e.g., Hopper et al., 2019; Ogden et al., 2009; Wikström-Grotell, 2016; World Physiotherapy, 2021)

In the literature, several authors have identified human movement as the principal phenomenon in physiotherapy discourse. Whereas Hislop (1975) claimed that human movement’s intra- and interpersonal levels rest on pathokinesiology, Tyni-Lenné (1988) has outlined a movement hierarchy based on the system theory perspective, and Cott et al. (1995) has defined human movement as occurring at multiple levels along a continuum, from the microscopic to the level of people acting in society. Several other authors have outlined the situational and contextual relatedness of human movement (e.g., Gentile, 1992; Gordon, 1987; Mulder, 1991). More recently, in the context of physiotherapy, human movement has been discussed as an aim, treatment, intervention, or an indicator of health (Broberg et al., 2003; Wikström-Grotell, 2016). Furthermore, a holistic view of human movement has also been highlighted, according to which an individual is an active agent, able to change and therefore, gain health and well-being to cope and fulfill their own meaningful goals in varying situations (Broberg et al., 2003; Wikström-Grotell, 2016). The need to understand human movement phenomena more deeply has also been highlighted (Sebelski et al., 2020; Wikström-Grotell, 2016).

Professional education has significant power to influence the quality of professions (Colby & Sullivan, 2008), and requires collaboration between the educational (academic) and clinical worlds in physiotherapy contexts (Jensen et al., 2017). Although physiotherapists’ unique use of human movement in practice has been recognized, a deeper understanding of learning and teaching human movement is still required (Sebelski et al., 2020). It has been acknowledged that learning and teaching human movement is at the heart of physiotherapy of physiotherapy clinical practice, and yet the literature has not addressed the depth and breadth of the knowledge or skills required for this (Sebelski et al., 2020; e.g.; Edwards et al., 2006; Zablotny et al., 2018). A deeper meaning of learning and teaching human movement, which may be unique to the profession, remains to be discovered in the context of physiotherapy (Jensen et al., 2017). Therefore, determining what is unique in learning and teaching human movement in physiotherapy requires further exploration, as does the way in which the topic is understood by physiotherapy students, therapists and instructors.

In the context of professional preparation, signature pedagogies are the characteristic form of the learning/teaching required in each field to prepare future practitioners for their profession (Shulman, 2005). The presumption is that “in these signature pedagogies, the novices are instructed in critical aspects of the three fundamental dimensions of professional work—‘to think, to perform, and to act’” (Shulman, 2005, p. 52). In the field of physiotherapy, education in professional practice and the supervisory processes it involves is an important, distinct part of entry-level education, and is also where the theories and knowledge of learning and teaching human movement are put into practice. As human movement has been identified as the core of physiotherapy education and practice, in physiotherapy, “signature pedagogy” thus entails “pedagogical signatures” related to learning and teaching human movement in both contexts. Therefore, to understand the phenomenon in more depth, learning and teaching human movement should be examined from the perspectives of all the stakeholders—students, therapists, and instructors—involved in education and practice (Parker et al., 0000). A synthesis of qualitative studies would illustrate how all these stakeholders understand and describe learning and teaching human movement in physiotherapy education and practice; this cannot be achieved through a synthesis of quantitative studies or a mixed-method systematic review (cf., Lockwood et al., 2020).

This qualitative systematic review and metasynthesis brings together primary qualitative research findings, appraised critically and synthesized with a set of new aims (Lockwood et al., 2020). To our knowledge, no qualitative systematic review has previously synthesized the findings regarding the perceptions of learning and teaching human movement among students, therapists, and instructors in the context of physiotherapy education and practice. We assumed that a synthesis of qualitative studies would lead to a more comprehensive understanding of learning and teaching human movement in the physiotherapy context. Accordingly, this review contributes to the synthesis of available evidence and has implications for physiotherapy education and practice. It aims to identify, critically appraise, and synthesize the best available research literature on learning and teaching human movement, as perceived by physiotherapy students, therapists, and instructors. Our research question was: How do students, therapists, and instructors perceive learning and teaching human movement in physiotherapy education and practice?

## Methods

This systematic review was conducted in accordance with the Joanna Briggs Institute (JBI) methodology for systematic reviews of qualitative evidence (Lockwood et al., 2020). Before commencing the study, we registered the review protocol in the PROSPERO database (registration number: CRD42020171836, submitted for registration 4 March 2020) and the JBI Systematic Review Register (registered 20 January 2021). At the time of registration, the preliminary search found no existing or current systematic reviews on this topic.

### Inclusion criteria

The above-mentioned research question guided the development of specific review criteria, which was based on the PICo mnemonic, i.e., Population (P), Phenomena of Interest (I), and Context (Co). The review included studies of physiotherapy students’, therapists’, and instructors’ (P) perceptions of learning and/or teaching human movement (I) in higher educational or clinical practice (Co) contexts. We use the term *student* in this review to refer to a student physiotherapist. By *therapist* we mean a physiotherapist working with patients/clients, and by *instructor* we refer to a physiotherapy teacher or physiotherapist who works with patients/clients and facilitates/supervises student learning.

#### Types of studies

This systematic review looked at studies that focused on qualitative data including, but not limited to, phenomenology, phenomenography, grounded theory, ethnography, and action research. The aim was to illustrate how physiotherapy students, therapists, and instructors understand and describe learning and teaching human movement, which cannot be done using quantitative methods (cf., Lockwood et al., 2020). However, studies that used mixed (qualitative and quantitative) methods were included if they presented their qualitative aims and results separately.

### Search strategy

This review had a three-step search strategy. First, we conducted an initial limited search of MEDLINE and CINAHL databases using the initial keywords. Second, we screened the titles and abstracts of the identified studies for keywords and identified the index terms used to describe the article. Third, we conducted the final search, which used all the identified keywords and the index terms, in all the selected databases. The systematic searches were conducted by an information specialist in consultation with the reviewers. The full search strategies, filters, and dates, and the different databases searches are described in Appendix 1.

The search spanned a range of sources for studies published from 1970 to 9 April 2020 in the following databases: MEDLINE (PubMed); CINAHL (EBSCO), ERIC, and PsycINFO, the Finnish medical bibliographic database MEDIC, and the Finnish archives database FINNA. We considered studies published in English, Finnish, and Swedish for inclusion. We conducted an updated search in the same six databases on 29 March 2022. The time period was considered the most relevant to current clinical or educational settings in physiotherapy. However, we were aware that electronic database availability only became more common from the 1990s onwards.

### Study selection

Following the search, we collated all the identified citations and uploaded them to RefWorks Version 2.0 (ProQuest), after which we removed duplicate citations. Two independent reviewers (SA, PV) screened the titles and abstracts for eligibility, using pre-determined inclusion criteria. Potentially relevant studies were retrieved as full texts, and their details were imported into the JBI System for the Unified Management, Assessment and Review of Information (Munn et al., 2019). Two independent reviewers (SA, PV) assessed the inclusion criteria of the full texts of the studies. They excluded any full-text studies that did not meet the inclusion criteria and recorded the reasons for exclusion. Any disagreement between the reviewers was resolved through discussion or with a third reviewer (Author 2). The search results were reported in full in the final systematic review and presented in a PRISMA flow diagram (Page et al., 2021).

### Assessment of methodological quality

The studies selected for inclusion in the review were critically assessed by two independent reviewers (SA, PV) using the standardized tool for qualitative research incorporated within JBI SUMARI (Lockwood et al., 2020). The same tool was also used for assessing the qualitative dimension of the mixed-method studies included in this review. This critical appraisal tool uses a series of criteria that can be scored on the basis of whether they are met or not met, unclear, or are not applicable to a particular study. Two independent reviewers (SA, PV) commented on each criterion, and any disagreements between them were resolved through discussion or with a third reviewer. One of the two reviewers was a JBI-certified reviewer. The reviewers first piloted the JBI Critical Appraisal tool on a study not included in this review. The critical appraisal results are presented in tabular form (Table 1). All the studies, regardless of the results of their methodological quality, underwent data extraction and synthesis. In line with Lockwood et al. (2020) no cut-off score for inclusion or exclusion was used in this systematic review.

**Table 1.** Critical appraisal results of included studies.

Study	Q1	Q2*	Q3*	Q4*	Q5	Q6*	Q7*	Q8	Q9	Q10	Total of Y/10
Ahola et al. (2017)	U	Y	Y	Y	Y	Y	N	Y	Y	Y	8/10
Covington and Barcinas (2017)	U	Y	Y	Y	Y	Y	Y	N	Y	Y	8/10
Denneny et al. (2020)	N	N	N	N	N	Y	Y	Y	Y	Y	5/10
Durham et al. (2009)	Y	Y	Y	Y	Y	Y	N	U	Y	Y	8/10
Embrey et al. (1996)	U	Y	Y	Y	Y	Y	N	Y	Y	Y	8/10
Embrey and Hylton (1996)	U	Y	Y	Y	Y	Y	N	Y	Y	Y	8/10
Farjoun et al. (2020)	U	Y	U	Y	Y	Y	Y	Y	Y	Y	8/10
Hedlund and Gyllensten (2013)	U	Y	Y	Y	Y	Y	N	U	Y	Y	7/10
Johnson et al. (2013)	U	U	Y	Y	Y	Y	Y	U	Y	Y	7/10
Lindvall et al. (2016)	U	Y	Y	Y	Y	Y	Y	U	Y	N	7/10
Normann et al. (2014)	Y	N	N	N	Y	Y	Y	N	Y	N	5/10
Ryan et al. (2020)	U	U	Y	Y	Y	Y	Y	N	Y	N	6/10
Skjaerven et al. (2010)	U	N	N	N	N	Y	Y	U	Y	Y	4/10
Skjaerven et al. (2008)	U	N	Y	N	N	U	Y	U	N	N	2/10
Stephenson and Stephens (2018)	U	Y	Y	U	Y	Y	Y	Y	Y	Y	8/10
Svensen and Bergland (2007)	U	N	U	U	Y	Y	Y	U	Y	N	4/10
Wohlin Wottrich et al. (2004)	U	Y	Y	Y	Y	Y	Y	U	Y	Y	8/10
%	11.11	61.11	72.22	66.66	77.77	94.44	66.66	33.33	94.44	72.22	

*ConQual dependability questions.

Y, Yes; N, No; U, Unclear; JBI Critical Appraisal Checklist for Qualitative Research (Lockwood et al., 2015).

Q1, Is there congruity between the stated philosophical perspective and the research methodology? Q2, Is there congruity between the research methodology and the research question or objectives? Q3, Is there congruity between the research methodology and the methods used to collect data? Q4, Is there congruity between the research methodology and the representation and analysis of data? Q5, Is there congruity between the research methodology and the interpretation of results? Q6, Is there a statement locating the researcher culturally or theoretically? Q7, Is the influence of the researcher on the research, and vice versa, addressed? Q8, Are participants, and their voices, adequately represented? Q9, Is the research ethical according to current criteria or, for recent studies, is there evidence of ethical approval by an appropriate body? Q10, Do the conclusions drawn in the research report flow from the analysis, or interpretation, of the data?

### Data extraction

Data were extracted from the papers selected for the review by the two independent reviewers (SA, PV), using the standardized data extraction tool in JBI SUMARI. All disagreements arising between the reviewers were resolved through discussion or with a third reviewer. In the first phase of the data extraction, study characteristics were extracted: study methods (data collection and analysis), geographical location, phenomena of interest, context, the participant characteristics and sample size, and the description of the main results. The second phase of the data extraction dealt with the specific study findings were extracted: physiotherapy students’, therapists’, and/or instructors’ perceptions of learning and/or teaching human movement in physiotherapy education or practice.

The reviewers also extracted illustrations from the text that supported these findings by one illustration per finding. The findings and illustrations were verbatim quotes from the study participants and authors. In line with Lockwood et al. (2020) the credibility of all the findings was assessed as unequivocal (the finding was accompanied by an illustration that was beyond a reasonable doubt and not open to challenge), credible (the finding was accompanied by an illustration lacking a clear association and open to challenge), or not supported (when neither an equivocal nor credible illustration could be applied and when the data did not support the most notable findings or when there were no illustrations to support the finding).

### Data synthesis

The study findings in this review of qualitative studies were pooled by two independent reviewers (SA, PV) using JBI SUMARI and the meta-aggregation methods (cf. Lockwood et al., 2015, 2020). This involved aggregating or synthesizing the findings to generate a set of statements to represent the aggregation assembling the findings and categorizing them on the basis of similarity in meaning. Finally, we synthesized these categories to produce a single comprehensive set of synthesized findings.

### Assessing confidence in the findings

The final synthesized findings were graded according to the ConQual approach for establishing confidence in the output of qualitative research synthesis and were presented in a ConQual summary of findings (see Munn et al., 2014), which we present at the end of the next chapter (Table 7). This summary included the significant elements of the review and details of how the ConQual score was developed. Each synthesized finding from the review was presented along with the type of research informing it, dependability and credibility scores, and the overall ConQual score.

## Results

### Study selection

**Figure 1.**
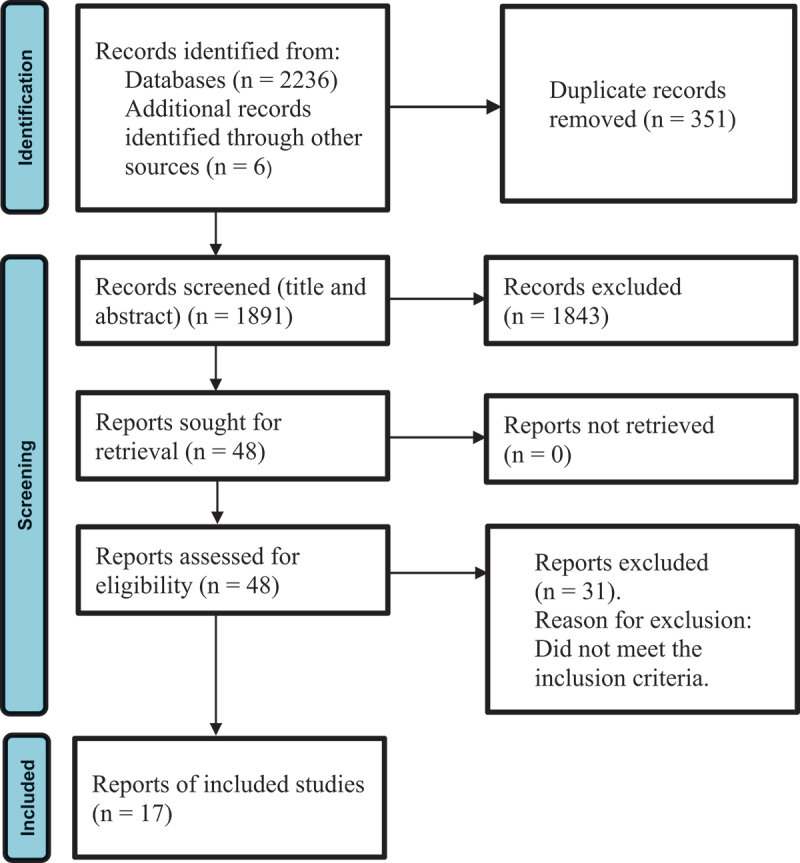
Search results and study selection and inclusion process (PRISMA Flow Diagram, see Page et al., 2021).

A comprehensive literature search of the databases returned 2236 records. In addition to this, we found six potential studies were found from the reference lists of the included studies. After removing the duplicates (*n* = 351), we assessed the titles and abstracts of the remaining records (*n* = 1891). A further 1843 records were excluded on the basis of title and abstracts screening, leaving 48 reports for full-text screening. The full-text screening led to 31 reports being excluded. The main reason for study exclusion was that the phenomena of interest were not compatible with the systematic review. No disagreements arose during the screening process. The remaining 17 reports were critically appraised and included in the review. Figure 1 presents the full study selection process.

## Methodological quality

Table 1 presents the critical appraisal results of the 17 studies selected for the review, based on the JBI Critical Appraisal Checklist for Qualitative Research. The methodological assessment revealed that the quality of the studies varied, the total scores of the individual studies being between 2 and 8, and none of the studies achieving the maximum score of 10. Eight of the studies achieved a score of 8; three studies scored seven; one study scored of 6; two studies scored 5, two studies scored 4; and one study scored 2. Only two studies met the quality criteria in Q1 (congruity between the stated philosophical perspective and research methodology), and six studies met those in Q8 (participants and their voices adequately represented). Seven studies did not meet the criteria in Q2 (congruity between the research methodology and the research question or objectives), and six studies did not meet those in Q7 (the influence of the researcher on the research and vice-versa). No study was excluded for reasons of quality.

With regard to the dependability questions for determining the ConQual score (Q2, Q3, Q4, Q6, Q7), three studies achieved a score of 5, nine studies achieved a score of 4, and the remaining five studies achieved a score of 2. Table 1 presents the results of the critical appraisal process.

### Characteristics of selected studies

The characteristics of the studies selected for the review are presented in Appendix 2. All the included studies (*n* = 17) used qualitative methods to investigate the perceptions of physiotherapy students, therapists and/or instructors with regard to learning and teaching human movement in physiotherapy and had been published in English. In 14 studies, ^3–15, 17^ the participants were therapists in the physiotherapy practice context; and in two studies, ^1,16^ the participants were entry-level physiotherapy students. In one study,^2^ the participants were both physiotherapy students and instructors. The perceptions of participants other than physiotherapy students, therapists and instructors were also presented in six of the selected 17 studies. Only the findings representing the perceptions of the physiotherapy students, therapists, and/or instructors related to learning and/or teaching human movement in physiotherapy, education and/or practice contexts were extracted from these and included in the analysis.

The sample sizes ranged from 4 to 15 participants in each study’s physiotherapy education or practice context. The total number of participants was 142. Twenty of these were physiotherapy students (female *n* = 11, male *n* = 4, gender not reported or sex not revealed *n* = 5) in the higher education, academic context. Of the remaining 122 participants (female *n* = 80, male *n* = 9, gender not reported *n* = 33), 117 were therapists working with patients/clients and 5 were instructors working as a therapist with patients/clients and facilitators/supervisors of student learning. The five instructors had 18.5 years of working experience. Ten of the 117 therapists were described as experienced physiotherapists and six as inexperienced or novice physiotherapists, and the remaining 101 therapists were described as physiotherapists with different lengths of physiotherapy work experience (3–39 years). None of the instructors were higher education physiotherapy teachers or educators.

Of the studies in the review, 14 focused on one main context: the academic Bachelor program in higher education,^1,16^ clinical practice internships,^2^ outpatient pain management, teaching hospital,^3^ neurological rehabilitation hospital,^4,9^ outpatient centres for children with cerebral palsy,^5,6^ the Association of Bobath-trained Therapists with paediatric speciality,^7^ the Institute of Basic Body Awareness Therapy,^8^ primary health care,^10^ regional hospital, outpatient clinics for people with MS, ^11^ or rehabilitation hospitals for children with cerebral palsy.^12^ In addition, of the 17 studies in this review, three involved the following several contexts: 1. neurology, primary health care, and mental health; ^13^ 2. neurology, primary health care and psychiatric/psychosomatic physiotherapy;^14^ and 3. neurological and geriatric rehabilitation units.^17^

The 17 studies selected for this review represented seven countries: Four were from the United Kingdom,^3,4,9,15^ four from Norway,^11,13,14,16^ three from the United States of America,^2,5,6^ three from Sweden^8,10,17^, and one each from Canada,^12^ Finland,^1^ and Spain.^7^ All studies were written in English, and were published between 1996 and 2020: Two were published in 1996^5,6^, eight were published between 2004 and 2014^4,8,9,11,13,14,16,17^, and seven were published between 2016 and 2020^1,2,3,7,10,12,15^.

### Review findings

The physiotherapy students’ and therapists’/instructors’ perceptions of learning and teaching human movement in physiotherapy, education, and practice contexts are represented by four synthesized findings (Appendix 3). None of the included studies examined the teacher perspective, and thus, the instructor perspective in the synthesized findings is represented by physiotherapists, whose perceptions of learning and teaching human movement were examined in either physiotherapy practice (as a physiotherapist working with patients/clients) or education (as an instructor, facilitator/supervisor of student learning).

We present a brief description of each category within the four synthesized findings. Every category contains at least one illustration of the physiotherapy students’ and/or therapists’/instructors’ original expressions of their perception (including experience, understanding, conception, attitude, view, or belief) of learning and teaching human movement. Synthesized findings 1 and 3 concern the perspectives of physiotherapy students and therapists/instructors, and synthesized findings 2 and 4 concern the perspectives of therapists/instructors.

The synthesized findings were aggregated from 17 categories, and 147 findings were identified in the 17 studies. The credibility of these study findings was assessed as either unequivocal (*n* = 33) or credible (*n* = 114) (Table 2). In addition, we extracted nine unsupported findings with or without illustrations from seven studies. The unsupported findings were not included in the meta-aggregation. Tables 4, 5 , 6 and 7 present the meta-aggregative tables with synthesized findings and categories. Below, we present the four synthesized findings and their categories.

**Table 2.** Credibility assessment of study findings.

Study	Unequivocal	Credible	Unsupported
Ahola et al. (2017)	4	4	1
Covington and Barcinas (2017)	0	5	0
Denneny et al. (2020)	0	10	0
Durham et al. (2009)	1	3	1
Embrey et al. (1996)	1	7	0
Embrey and Hylton (1996)	2	0	0
Farjoun et al. (2020)	1	8	0
Hedlund and Gyllensten (2013)	1	14	0
Johnson et al. (2013)	10	3	1
Lindvall et al. (2016)	0	6	0
Normann et al. (2014)	0	7	0
Ryan et al. (2020)	4	4	0
Skjaerven et al. (2010)	0	17	3
Skjaerven et al. (2008)	5	11	0
Stephenson and Stephens (2018)	2	9	1
Svensen and Bergland (2007)	1	3	1
Wohlin Wottrich et al. (2004)	1	3	1
Total findings:	33	114	9

### Synthesized finding 1: Perceived significance of being present in movement

The physiotherapy students and therapists/instructors perceived that being present in movement was central to learning and teaching human movement in the context of physiotherapy education and practice. The first synthesized finding had three categories (Table 3). The first two categories illustrate the perceived significance of being present in movement per se, and being present in movement in relation to oneself, others, and the environment, from a physiotherapy student perspective. The third category illustrates the perceived significance of being present in movement as perceived by therapists/instructors of physiotherapy. These three categories are based on 27 findings of six studies^1,8,10,13,14,16^ (see Appendix 2). These findings were evaluated as either unequivocal (*n* = 5) or credible (*n* = 22).

**Table 3.** Synthesized finding 1: Perceived significance of being present in movement.

Categories	Synthesized finding
1.1 Perceived significance of being present in movement per se from students’ perspective *Students acknowledged that being present in movement per se was central to learning and teaching movement*	1. Perceived significance of being present in movement *Students and therapists/instructors perceived that being present in movement was central to learning and teaching movement*
1.2 Perceived significance of being present in movement in relation to oneself, others, and the environment *Students expressed that being present in movement in relation to oneself, others, and the environment was central to learning and teaching movement*	
1.3 Perceived significance of being present in movement from a therapist’s perspective *Therapists/instructors acknowledged that being present in movement was central to learning and teaching movement*	

#### Category 1.1: Perceived significance of being present in movement per se from students*’* perspective

The physiotherapy students perceived that listening and encountering different movement sensations and learning to trust their own bodily sensations was generally significant in terms of being present in movement. They also highlighted the significance of learning from their bodily sensations and what they found comfortable or discomfortable in the movement.

*I thought it was important to be aware of my body, to feel certain movements … and I also learned about myself and my limitations, what I was comfortable with and what I wasn’t comfortable with (p. 42)* (Svensen & Bergland, 2007)

#### Category 1.2: Perceived significance of being present in movement in relation to oneself, others, and the environment

The students perceived being present in movement as challenging but significant in terms of becoming more aware of the situation in relation to oneself, others, and the environment, for learning and teaching human movement. They stressed the significance of learning to put oneself in the other’s position.

*I do see the point, many of the movements were strange and awkward, but you have to go through it to be able to put yourself in someone else’s situation. (p. 43)* (Svensen & Bergland, 2007)

#### Category 1.3: Perceived significance of being present in movement from a therapist’s perspective

The therapists/instructors described the significance of being present in human movement in terms of learning to listen to one’s bodily sensations and to become more aware of movement. They also emphasized the significance of being present in movement for facilitating communication and encountering other people.

*You start to listen more to signals from the body and you can speak up more easily and then you dare to invite others. (p. 174)* (Hedlund & Gyllensten, 2013)

### Synthesized finding 2: Perceived significance of movement quality

This second synthesized finding revealed that the therapists/instructors perceived understanding and guiding movement quality as central in learning and teaching human movement. The second synthesized finding had three categories (Table 4) based on 24 findings of six studies ^4,5,7,10,13,14^ (see Appendix 2). The findings were evaluated as either unequivocal (*n* = 6) or credible (*n* = 18).

**Table 4.** Synthesized finding 2: Perceived significance of movement quality.

Categories	Synthesized finding
2.1 Perceived significance of guiding movement quality *Therapists/instructors acknowledged that guiding movement quality was central to learning and teaching movement*	2. Perceived significance of movement quality *Therapists/instructors perceived that understanding and guiding movement quality was central to learning and teaching movement*
2.2 Perceived significance of understanding the meaning of movement quality *Therapists/instructors expressed that understanding the meaning of movement quality was central to learning and teaching movement*	
2.3 Perceived significance of different perspectives of movement quality *Therapists/instructors expressed that the different perspectives of movement quality were significant in learning and teaching movement*	

Next, we elaborate on the significance of movement quality as described by therapists/instructors in relation to movement quality guidance, understanding the meaning of movement quality, and the various perspectives of movement quality.

#### Category 2.1: Perceived significance of guiding movement quality

The therapists/instructors described the significance of guiding movement quality in relation to promoting optimal movement. They emphasized how important guiding movement quality is for helping learners to move “correctly”. This indicated that therapists/instructors considered finding a “normal” or “typical movement” pattern while aiming to “avoid pathological patterns” possible. When describing movement as a “pathological movement pattern”, for example, they considered compensative movements undesirable and obstacles to “normal” [or typical] movement.

*they are moving so they are moving it correctly rather than learn the wrong way to do it (p. 84)* (Durham et al., 2009)

*I try to give symmetry, reduce compensations, a normalized posture, trying to avoid the presence of the postures that we do not like, that is, pathological patterns (P4) (p. 7)* (Farjoun et al., 2020)

#### *Category 2.2*: Perceived significance of understanding the meaning of movement quality

The therapists/instructors described the significance of understanding the meaning (or importance) of movement quality as being related to how the person moves and their movement expression and integration, and to discovering new ways of moving.

*Movement quality is seeing how the whole person moves; how the movements are coordinated; the timing. When you have the quality, you are steady, you involve the whole body, from the toes to the top, even the mimic and gestures are integrated, you have enough muscle tone and you observe that everything is in place. (p. 20)* (Skjaerven et al., 2008)

*The person needs to practice to learn details in a slow tempo first in order to keep the same level of integration in a faster speed. When the movement is integrated, it becomes harmonious. (p. 1486)* (Skjaerven et al., 2010)

#### Category 2.3: Perceived significance of different perspectives of movement quality

The therapists/instructors described the significance of learning to understand different perspectives of movement quality. They perceived movement quality as reflecting qualitative, environmental, and individual aspects of movement, i.e., biomechanical (path and form), physiological (flow, elasticity, rhythm), psychosocial/cultural (intention, emotion), and existential perspectives. They also highlighted the significance of recognizing preconditions, such as awareness of vertical axis and breathing, for learning and teaching movement quality.

*How the breathing is, effect the movement quality … if the breathing is with-held, it colors the movement quality. If you experience freedom in the movements, it affects the breathing; there is such a mutual interplay (p. 18) It is interesting to see movement quality in relation to culture … Movement quality comes from the interplay between the person and the environment. (p. 19)* (Skjaerven et al., 2008)

### Synthesized finding 3: Perceived significance of movement transfer

The physiotherapy students and therapists/instructors perceived that the enhancement of movement transfer was central to finding optimal ways of promoting acceptance, promoting learner engagement, modifying movement, varying the ways of guiding movement, and enhancing self-confidence in learning and teaching human movement in physiotherapy education and practice contexts. The third synthesized finding had seven categories (Table 5) based on 71 findings from all the 17 studies^1–^^17^(see Appendix 2). The findings were evaluated as either unequivocal (*n* = 21) or credible (*n* = 50).

**Table 5.** Synthesized finding 3: Perceived significance of movement transfer.

Categories	Synthesized finding
3.1 Perceived significance of finding optimal ways to enhance movement transfer *Students and therapists/instructors expressed that finding optimal ways to enhance movement transfer was significant in learning and teaching movement*	3. Perceived significance of movement transfer *Students and therapists/instructors perceived that enhancement of movement transfer was central in learning and teaching movement*
3.2 Perceived significance of promoting the acceptance of movement-related bodily perceptions *Therapists/instructors expressed that promoting the acceptance of movement-related bodily perceptions was significant in learning and teaching movement*	
3.3 *Perceived significance of promoting learner engagement promotion* *Therapists/instructors expressed that promoting learner engagement was significant in learning and teaching movement*	
3.4 Perceived significance of tailored movement modification *Therapists/instructors expressed that tailored movement modification was significant in learning and teaching movement*	
3.5 Perceived significance of using the most appropriate guidance for achieving the intended goal *Therapists/instructors expressed that using the most appropriate guidance for achieving the intended goal was significant in learning and teaching movement*	
3.6 Perceived significance of varying the guidance of movement *Therapists/instructors expressed that varying the guidance of movement was significant in learning and teaching movement*	
3.7 Perceived significance of enhancing self-confidence *Therapists/instructors expressed that enhancing self-confidence was significant in learning and teaching movement*	

#### Category 3.1: Perceived significance of finding optimal ways to enhance movement transfer

The physiotherapy students and therapists/instructors perceived that constantly considering and discussing optimal ways and learning to identify the most beneficial ways was significant for movement transfer enhancement. They also highlighted the significance of the learning process through observing, experiencing, and practicing, either with guidance or independently, and implementing it in everyday life.

*observing in the beginning, but then wanted to get her hands on and practice with guidance from me. And then transitioned to not only independent handling skills, but teaching the handling skills to the family as part of the home program (p. 607)* (Covington & Barcinas, 2017)

#### Category 3.2: Perceived significance of promoting the acceptance of movement-related bodily perceptions

The therapists/instructors perceived that focusing on healthy movement aspects, encouraging the learner’s own movement experience, and learning to guide the learner’s own choices of movement was significant when trying to promote the acceptance of movement-related bodily perceptions. They also highlighted the significance of learning that the same end can be achieved by different means when promoting the acceptance of movement-related bodily perceptions.

*D: When they’re moving and stretching and they hear clicking it can be quite frightening, so it’s just to remind them these are normal sounds … it’s to remind them that it’s kind of normal. (p. 166)* (Denneny et al., 2020)

*How about we set up an experiment or how about you give an alternative approach and give this a go and just see what happens, and it might be that your way is a better way, but you might like the new way. (p. 169)* (Denneny et al., 2020)

#### Category 3.3: Perceived significance of promoting learner engagement

The therapists/instructors perceived that emphasizing an inspiring experience at the beginning and exploring a genuine, personally meaningful goal is significant for promoting learner engagement in movement. They also stressed teaching the significance of movement to personally benefit the client.

*[I] see what sparks them, see when they start to pay more attention to what I’m saying, see what their language is and what they want to talk about. I really watch and listen and try to go with their lead … (Brigette) (p. 84)* (Ryan, Wright, and Levac, Ryan et al., 2020)

#### Category 3.4: Perceived significance of tailored movement modification

The therapists/instructors perceived that searching for and offering options, and constantly learning, and considering changes was significantly related to movement modification. They also emphasized the significance of patience, sensitivity, and acceptance of confusing experiences.

*I did not feel I got any results beyond the actual situation … I feel like having an “empty head” and “empty hands” so to speak (A) (p. 30)* (Normann et al., 2014)

*But then, suddenly…The patient does not say so much but shows up every week. Something has started, which was felt deeply and genuinely inside. (p. 173)* (Hedlund & Gyllensten, 2013)

*Category 3.5: Perceived significance of using the most appropriate guidance for achieving the intended goal*

The therapists/instructors perceived the significance of attaining the targeted function and learning to use the most appropriate guidance for achieving the intended goal. They also highlighted the significance of directing attention towards resources and functional purpose in movement.

*First of all, I try to examine their motor capacity, where the resources are … (PT) (p. 1200)* (Wohlin Wottrich et al., 2004)

*To be functional I guess, he’s not going to be thinking about his arm, where it is and how it feels, he is going to be thinking about what he is achieving (p. 85)* (Durham et al., 2009)

#### Category 3.6: Perceived significance of varying the guidance of movement

The therapists/instructors perceived that varying and combining internal (i.e., receiving sensory feedback through movement) and external (i.e., verbal, visual or physical) ways of guiding, and progressing according to the learner’s response, was significant for learning and teaching movement. They also highlighted the significance of giving the learner enough time and opportunity to receive feedback from internal sensations in movement learning.

*take away some of my support … have them do an activity without any guidance … take away … or vary my amount of assistance. (Brigette) (p. 86)* (Ryan, Wright, and Levac, 2020)

*Moving is an interplay between internal and external references; both are necessary for the movement to be functional and the training to be effective. I choose between the two, depending on the patient’s response. I offer time and opportunity for the patient to receive feedback from inside (p. 1488)* (Skjaerven et al., 2010)

#### Category 3.7: Perceived significance of enhancing self-confidence

The therapists/instructors perceived enhancing self-confidence as significant for learning and teaching movement. They described that learners’ autonomy gradually increased when they received positive feedback, that their dependence on supervision decreased, and their own decision-making was supported. They also described how facilitating learner reflection in different situations was essential for improving self-understanding.

*The patients start to reflect about what is happening and through reflecting over what goes on in the body in different situations, at home or where ever they are. Then they spontaneously start to reflect (p. 172)* (Durham et al., 2009)

*C: “The ultimate aim is for them to move with confidence rather then moving in a way that we want them to move.”(p. 169)* (Denneny et al., 2020)

### Synthesized finding 4: Perceived significance of contextual factors

The therapists/instructors perceived contextual factors such as the environment, staff resources, family members, and therapists as significant in learning and teaching movement. The fourth synthesized finding had four categories (Table 6) based on 26 findings in 12 studies^2–7,^^11–15,17^ (see Appendix 2). These findings were evaluated as either unequivocal (*n* = 1) or credible (*n* = 25).

**Table 6.** Synthesized finding 4: Perceived significance of contextual factors.

Categories	Synthesized finding
4.1 Perceived significance of environment-related contextual factors *Therapists/instructors expressed that acknowledgement of environment- related contextual factors was central to learning and teaching movement*	4. Perceived significance of contextual factors *Therapists/instructors perceived that acknowledgement of contextual factors was central to learning and teaching movement*
4.2 Perceived significance of human resource-related contextual factors *Therapists/instructors expressed that consideration of human resource- related factors was central to learning and teaching movement*	
4.3 Perceived significance of family member-related contextual factors *Therapists/instructors expressed that consideration of family member- related factors was central to learning and teaching movement*	
4.4 Perceived significance of therapist-related factors *Therapists/instructors expressed that consideration of therapist-related factors was central to learning and teaching movement*	

#### Category 4.1: Perceived significance of environment-related contextual factors

The therapists/instructors perceived that encouraging people to return to former functions and learning to take physical environmental factors into account were significant for environment-related contextual factors. They also highlighted the significance of the familiar domestic environment and exploring in silence for new experiences of learning and teaching movement.

*It is important to regain as many of the old functions as possible, to come back to one’s usual role, so it was important for her to go home. (PT) (p. 1202)* (Wohlin Wottrich et al., 2004)

*As [a] physical therapist, you teach the patient the process of exploring and searching—it is basic for making new movement experiences. The exploration is in itself important for the learning when it is done in silence. (p. 1486)* (Skjaerven et al., 2010)

#### Category 4.2: Perceived significance of human resource-related contextual factors

When highlighting the significance of human resource-related factors, the therapists/instructors emphasized time management: for example, freeing up time for human resources by using robotic-based treatment. They also emphasized the usefulness of group therapy for the appropriate use of human resources.

*It’s fine to do it in a group, it doesn’t need to be one to one … as long as they’re all getting enough attention. (Penny) (p. 249)* (Stephenson & Stephens, 2018)

*Category 4.3: Perceived significance of family member-related contextual factors*

When speaking about teaching and guiding a child’s movement, the therapists/instructors emphasized the significance of family member-related factors. They saw it as essential to take into account not only the child’s motor impairment, functional capacity, personal circumstances, and personality, but also the family members’ preferences and interests.

*If you forget how the child’s way to school is going, that the mother needs a tool so that she can feed her, that she cannot be there 24 hours a day with an adult who corrects her (p. 8)* (Farjoun et al., 2020)

#### Category 4.4: Perceived significance of therapist-related factors

In terms of therapist-related factors the therapists/instructors perceived that being open and nonjudgmental, and learning to trust and accept, is significant in learning and teaching human movement. In addition, they emphasized the importance of learning from others and enduring uncertainty. They also stressed the significance of the therapist/instructor’s awareness of their own movement.

*I communicate movement through being in movement and by being in rhythm. I influence the patient through my own closeness to movement (p.1484) I must accept what happens … Trust and acceptance are psychological aspects important for bringing therapy forward. (p.1485)* (Skjaerven et al., 2010)

### Summary of review findings

The final synthesized findings were graded using the ConQual approach to establish confidence in the output of qualitative research synthesis (Munn et al., 2014) and were presented in the ConQual summary of findings (Table 7). Each qualitative research finding, i.e., i) being present in movement; ii) movement quality; iii) movement transfer, and iv) contextual factors, was assigned a level of dependability and credibility. The overall quality of the studies scored low on ConQual, and dependability and credibility were rated as moderate.

**Table 7.** ConQual summary of findings.

Perceptions of learning and teaching human movement in physiotherapy: A systematic review and metasynthesis of qualitative studies					
Synthesized findings	Type of research	Dependability	Credibility	ConQual score	Comments
1. Perceived significance of being present *Students and therapists/instructors perceived that being present in movement was central to learning and teaching movement*	Qualitative	Moderate (downgraded one level*)	Moderate (downgraded one level**)	Low	Dependability: downgraded due to the dependability of the (6) primary studies: one study addressed five, two addressed four and three addressed two of the five dependability questions. Credibility: downgraded due to a mix of unequivocal and credible findings (5 U, 22C).
2. Perceived significance of movement quality *Therapists/instructors perceived that understanding and guiding movement quality was central to learning and teaching movement*	Qualitative	Moderate (downgraded one level*)	Moderate (downgraded one level**)	Low	Dependability: downgraded due to the dependability of the (6) primary studies: one study addressed five, three addressed four and two addressed two of the five dependability questions. Credibility: downgraded due to a mix of unequivocal and credible findings (6 U, 18C).
3. Perceived significance of movement transfer *Students and therapists/instructors perceived that enhancement of movement transfer was central in learning and teaching movement*	Qualitative	Moderate (downgraded one level*)	Moderate (downgraded one level**)	Low	Dependability: downgraded due to the dependability of the (17) primary studies: three studies addressed five, nine addressed four and five addressed two of the five dependability questions. Credibility: downgraded due to a mix of unequivocal and credible findings (20 U, 45C).
4. Perceived significance of contextual factors *Therapists/instructors perceived that acknowledgement of contextual factors was central to learning and teaching movement*	Qualitative	Moderate (downgraded one level*)	Moderate (downgraded one level**)	Low	Dependability: downgraded due to the dependability of the (12) primary studies: three studies addressed five, six addressed four and three addressed two of the five dependability questions. Credibility: downgraded due to a mix of unequivocal and credible findings (1 U, 25C).

U, unequivocal; C, credible.

*Downgraded due to a mix of high and moderate dependability scores.

**Downgraded due to a mix of credible and unequivocal findings.

## Discussion

This systematic review aimed to explore the qualitative evidence related to students’, therapists’, and instructors’ perceptions of learning and teaching human movement in physiotherapy education and practice. The systematic review identified no studies from the perspective of teachers. Therefore, the instructor perspective was represented by physiotherapists supervising student learning in practice. Altogether, the review synthesized 142 participants’ perceptions of the phenomenon of interest, from the perspectives of students (*n* = 20) and therapists/instructors (*n* = 122). It comprised 17 studies, and resulted in 33 unequivocal and 114 credible findings, which were grouped into 17 categories.

From the meta-aggregation of the selected studies, we identified the following four synthesized findings presenting physiotherapy students’ and therapists/instructors’ perceived significance of learning and teaching human movement in physiotherapy educational and practice contexts: i) being present in movement; ii) movement quality; iii) movement transfer, and iv) contextual factors. Two of the synthesized findings, namely “being present in movement” and “movement transfer” illustrated the perspectives of physiotherapy students and therapists/instructors, whereas the remaining two synthesized the findings of “movement quality” and “contextual factors” related to the therapist/instructor perspective only. We now discuss each of the synthesized findings separately.

The first synthesized finding described the significance of “being present in movement” for learning and teaching human movement in physiotherapy. The extracted findings show that the students and the therapists/instructors acknowledged that being present had a two-fold meaning related to movement and the self. The students in particular highlighted the importance of being present in terms of their own movement, but also in terms of others’ movement, especially when guiding the movement of others. The therapists/instructors in turn, although also highlighting the importance of being present in terms of their own movement, further highlighted the importance of their own movement communication when guiding and encountering others. The students also perceived learning and teaching human movement as a challenging but ongoing meaningful process.

The first synthesized finding in this review resonates with those of previous studies: Learning to be present in one’s own movement is challenging (Blackburn & Price, 2007; Todes, 2001) but essential for understanding and guiding the bodily experience of others (Engelsrud et al., 2019). These findings are concurrent with those of previous studies that have emphasized how being present in one’s own and one’s client’s movement requires “a dual state of being present” (Dewey, 1938; Rogers, 1989). According to Blackburn and Price (2007), the two important fundamental elements of physiotherapy for professionals’ “dual state of being present” are their own learning to be present in movement and encouraging the client to be present in movement. Our findings also resonate with those of previous studies (Chowdhury & Bjorbækmo, 2017; Engelsrud et al., 2019; Piirainen, 2006) that have emphasized how being present in movement requires awareness of non-verbal communication and guidance in physiotherapy.

The second synthesized finding of the review manifests a less documented significance of movement quality related to the therapist/instructor perspective of learning and teaching human movement in physiotherapy. In this review, therapists/instructors described the significance of movement quality in relation to three categories: movement quality guidance, understanding the meaning of movement quality, and the various perspectives of movement quality. Despite the acknowledged essential role of movement quality in the field, van Dijk et al. (2017) have previously reported a lack of knowledge of the way in which physiotherapists understand and describe the phenomenon of movement quality, which the results of this review aim to offer.

This review revealed that therapists/instructors acknowledged the importance of guiding movement quality to promote optimal movement capacity from the very beginning of the therapy/learning process. This resonates with a vision statement in the physiotherapy profession that asserts that in physiotherapy, optimal movement and functional capacity are core aims when promoting a client’s ability to engage with their daily environment (American Physical Therapy Association, Engelsrud et al., 2019). However, the studies included in this review also demonstrated a desire for “normal” or “correct” patterns of movement, as articulated by the therapists/instructors. It is noteworthy that the reviewed studies did not critically discuss the dated understanding of “normal” or “correct” patterns of movement, although the issue has been raised in previous literature (Nicholls & Vieira, 2022).

The third synthesized finding related to movement transfer conveys the significance of genuinely meaningful and person-centred goal-setting for learning and teaching human movement in physiotherapy. This is supported by previous studies highlighting the acknowledgement of learner motivation (E. H. Simpson & Balsam, 2016; cf.; Kiresuk et al., 1994) and personal values (Kangasniemi et al., 2015). The therapists/instructors in the included studies highlighted the significance of self-confidence in learning and teaching movement. They also emphasized the significance of sensitivity towards the manifestations of confusing movement sensations and experiences. These findings are concurrent with those of previous studies that highlight problems related to overly self-critical attitudes, and varying the therapist’s or instructor role according to the situation and needs of the learner (cf. Leiman, 2012; Piirainen, 2006).

The third synthesized finding resonates with those of previous studies that have highlighted the significance of the learner experience as a basis for teaching (Dewey, 1938; Klemola, 2004; Piirainen, 2006). Paying attention to bodily movement sensations may thus offer opportunities for experiences and learning (Klemola, 2004). In physiotherapy, the pedagogical relationship aims to help the client make sense of their problem and gradually learn to see and find ways in which to take charge of their situation (Piirainen, 2006). Similarly, it shows that through reflective communication with others, students can make connections between the professional curriculum and their real-world experiences. Critical reflection allows students to create new meanings from their experiences (Wright & Lundy, 2012), also when they are learning or teaching human movement in physiotherapy education or practice.

The fourth synthesized finding conveyed the therapists’/instructors’ perceived importance of taking the learner (i.e., student or patient/client), learning/teaching environment, and family, therapist/instructor, and other human-related contextual factors into account when learning and teaching human movement in physiotherapy. This synthesized finding is concurrent with previously described broad understandings of contextual factors as environmental factors, products and technology, the natural environment, social support and relationships, attitudes, and the availability of services (Tomey & Sowers, 2009). The synthesized finding also manifested the therapist/instructor-related significance of being open and nonjudgmental, and learning to trust and accept uncertainty in learning and teaching movement. Learning to deal with uncertainty has previously been identified as one of the most significant aspects of professionalism, which Shulman (2005) named “signature pedagogies of uncertainty”. Accordingly, Engelsrud, Nordtug, and Qien (2019), for example, have highlighted the significance of being present in the actual situation as a value for physiotherapists, as opposed to focusing on uncertainties related to goal achievement.

The fourth synthesized finding also conveyed the perceived significance of the therapists’ ongoing dialogue with the self and others through *being* in movement. An earlier study similarly illuminated the significance of an intuitive understanding of others, which arises as the result of bodily presence (Fuchs, 2016).

Overall, the synthesized findings of this review indicate that learning and teaching human movement and how it is perceived take centre stage in physiotherapy, education, and practice. The synthesized findings illuminate the perceived significance of contextual factors, movement transfer, movement quality, and being present in movement for learning and teaching movement, regardless of whether the context is physiotherapy education or practice. As such, these four synthesized findings can be discussed in terms of Shulman’s (2005) signature pedagogy and its significance for early socialization into the practice and values of a particular professional field. According to Shulman (2005), signature pedagogies prefigure the characteristic form of learning/teaching required to prepare future practitioners for their profession, whether it relates to learning and teaching in academic or in clinical settings. Furthermore, in signature pedagogies, novices are taught the critical aspects of the fundamental dimensions of professional work, “to think, to perform, and to act” (Shulman, 2005). Therefore, based on the results of this review, we suggest that novices in physiotherapy education and practice should be taught about the four synthesized findings related to learning and teaching human movement.

As such, the first synthesized finding embodies a dual awareness of the meaning of “presence” in relation to “movement” and “self”, followed by the second synthesized finding, which manifests significance of “movement quality”. When reflected from the perspective of Shulman’s (2005) pedagogical signatures in the context of physiotherapy education and practice, the first and the second synthesized findings illuminate the significance of “being present in movement” and “movement quality” when learning and teaching human movement. Accordingly, the third and fourth synthesized findings manifest the significance of acknowledging “movement transfer” and “contextual factors”, in physiotherapy learning and teaching human movement.

### Strengths and limitations

The strength of this review was that by conducting an extensive literature search and applying rigorous JBI methods, we were able to synthesize 142 informants’ perceptions of learning and teaching human movement in the context of physiotherapy education and practice. We chose the study aim on the basis of both an identified evidence-base knowledge gap and our personal interests as educators in higher education. All the authors have professional and research expertise in the field of health sciences and qualitative research; two are highly experienced experts and one is an advanced researcher. One of the experts is also trained in the JBI method. The authors’ backgrounds in the field of physiotherapy vary in both clinical and educational contexts such as health promotion, paediatrics, mental health, and neurological physiotherapy.

Another strength of this study is that the protocol was registered and made publicly available before the systematic review began. In addition, during the meta-aggregation process, the authors avoided any reinterpretation of original findings, but accurately represented the extracted findings from the studies included in this review. Furthermore, two of the reviewers completed the article screening, quality appraisal, and data extraction steps independently. The whole research group participated in piloting the quality appraisal tool.

In addition to the qualitative studies, this review also included mixed-method studies that reported qualitative results separately. A critical assumption of JBI methodology is that different methodologies can be mixed in a single synthesis of qualitative studies if they focus on the same phenomena of interest (Lockwood et al., 2020). However, the proportion of high-quality studies was low in this review, and the level of the synthesized evidence was weak due to the weaknesses in the methodological quality of the selected studies. In terms of qualitative research, this is a significant issue, especially for systematic reviews and meta syntheses. It is also important to note that qualitative research appraisal, as well as what has been considered important in reporting, has varied in different eras (Denzin & Lincoln, 2018).

A limitation of this study is that although the review’s studies consisted of 142 participants, 20 student physiotherapists, and 122 therapists/instructors, none of the participants were physiotherapy educators/teachers from higher education context. Thus, the findings of this review showed that the evidence on how teachers/educators in physiotherapy education perceive learning and teaching human movement is limited. This is a matter of great importance for future research, considering how central learning and teaching human movement is to physiotherapy higher education.

A challenge in this systematic review was its search strategy. In order to find studies that involved the phenomena of interest of this review, we used a broad search strategy with wide- ranging search terms and had help from an experienced information specialist. The search strategy aimed to find studies published in only English, Swedish, or Finnish, which is a potential limitation of this study.

To add to the trustworthiness of this study, we conducted an updated search of electronic databases MEDLINE, CINAHL, ERIC, PsycINFO, MEDIC, and FINNA on 29 March 2022, which yielded 548 studies, five of which were potential. We also found two potential studies through a manual search. Six of the studies were from a clinician’s perspective and addressed the following themes: movement quality in autism (Bertilsson et al., 2022), the vocabulary of movement quality (Skjaerven et al., 2020), wearable devices (L. A. Simpson et al., 2021; Vaughan-Graham et al., 2020), children with cerebral palsy (Storvold & Jahnsen, 2021), and physiotherapists’ movement awareness (Ahola et al., 2022). In addition, one study was of a physiotherapy student and addressed a specific approach to the movement awareness learning domain (BBAT) of physiotherapy (Bravo et al., 2022) in the educational context. The fact that these studies were not included in this review was a potential limitation. However, the updated search studies did not seem to contradict the core findings of this study; in fact, they supported its core findings.

Despite these weaknesses, the studies selected for this review made valuable contributions to the findings. Regardless of whether the stage was that of education or professional development, one of the enduring core phenomena encountered in physiotherapy seemed to be learning and teaching human movement. Direct descriptions of how learning and teaching human movement is perceived by physiotherapy students and therapists/instructors remain rare, and thus require further attention in future studies.

## Conclusion

The synthesized findings indicate the perceived significance of contextual factors, movement quality and transfer, as well as being present in movement, when learning or teaching human movement in physiotherapy. These findings can be seen as central content themes, which, according to this review, are significant and should be considered pedagogical signatures, in physiotherapy education and practice and the continuing education of physiotherapists/instructors. However, the overall ConQual quality score of the studies was low, and dependability and credibility were moderate (Table 7). This needs to be considered when applying these results in physiotherapy education or practice. The findings of this review also suggest that research of learning and teaching human movement in physiotherapy from the perspective of higher education teachers is lacking. This is a matter of great importance, both pedagogically and professionally, and requires further research.

### Recommendations for physiotherapy education and practice

Our recommendations for education and practice are based on the synthesized findings of this review. All the recommendations have been graded according to the JBI Grades for Recommendations (Lockwood et al., 2020) on level C (Table 7).

Recommendation 1 (combining synthesized findings 1 and 2): The concepts of “being present in movement” and “movement quality” should be integrated into learning and teaching human movement in physiotherapy education and practice.

Recommendation 2 (combining synthesized findings 3 and 4): The significance of “movement transfer” and “contextual factors” should be acknowledged when learning and teaching human movement in physiotherapy education and practice.

## Appendix

### Appendix 1. Search strategy databases MEDLINE, CINAHL, ERIC, PsycINFO, FINNA and MEDIC

**Table:**

Database	Search strategy:
MEDLINE	Search 25 March 2020
	((physical therapist* or physiotherapist* or physical therap* or physiotherap* or Undergraduate or pre-registration or post-registration or postgraduate or baccalaureate or diploma or continuing education or pre- licensure or post-licensure or student or teacher or supervisor or clinical instructor or educator or higher education or continuing education or clinical setting or clinical placement or practice placement or internship or practicum or healthcare facility) and (perceptions or experiences or conceptions or attitudes or feelings or views or beliefs) and (human movement or movement or movement quality or human movement quality or human movement embod* or movement embod* or movement phenomena or human movement phenomena or movement phenomenon or human movement phenomenon or movement perspective or human movement perspective or movement element or human movement element or movement process or human movement process or movement aspect or human movement aspect or movement communication or human movement communication or movement dialogue or human movement dialogue or movement description or human movement description or movement vocabulary or human movement vocabulary or movement awareness or human movement awareness or movement sensation or human movement sensation or movement control or human movement control or movement skill or human movement skill or movement knowledge or human movement knowledge or movement knowing or human movement knowing or human movement pedagog* or movement pedagog*) and (learning or learn* or reflect or assimilate or adopt or understand or develop or espouse or enhance or facilitate or teaching or teach* or train or promote or educate or support or guide or coach or instruct or supervise) and (qualitative research or qualitative or qualitative study or phenomenology* or phenomenograph* or grounded theory or qualitative* or content analysis or thematic analysis or interview* or focus group* or interpretat* or descriptive* or narrative* or ethnograph* or mixed method)).af. Filter: publication year from 1970 onwards
CINAHL	Search 27 March 2020
	(”physical therapist*”OR physiotherapist* OR “physical therap*” OR physiotherap* OR Undergraduate OR pre-registration OR post-registration OR postgraduate OR baccalaureate OR diploma OR “continuing education” OR pre-licensure OR post-licensure OR student OR teacher OR supervisor OR clinical instructor OR educator OR “higher education” OR “continuing education” OR “clinical setting” OR”clinical placement” OR “practice placement” OR internship OR practicum OR “healthcare facility”) AND (perceptions OR experiences OR conceptions OR attitudes OR feelings OR views OR beliefs) AND (“human movement” OR movement OR”movement quality” OR “human movement quality” OR “human movement embod*” OR “movement embod*” OR “movement phenomena” OR “human movement phenomena” OR “movement phenomenon” OR”human movement phenomenon” OR “movement perspective” OR “human movement perspective” OR “movement element” OR “human movement element” OR “movement process” OR “human movement process” OR “movement aspect” OR “human movement aspect” OR “movement communication” OR “human movement communication” OR “movement dialogue” OR “human movement dialogue” OR “movement description” OR “human movement description” OR “movement vocabulary” OR “human movement vocabulary” OR “movement awareness” OR “human movement awareness” OR “movement sensation” OR “human movement sensation” OR “movement control” OR “human movement control” OR” movement skill” OR “human movement skill” OR “movement knowledge” OR “human movement knowledge” OR “movement knowing” OR “human movement knowing” OR “human movement pedagog*” OR “movement pedagog*”) AND (learning OR learn* OR reflect OR assimilate OR adopt OR understand OR develop OR espouse OR enhance OR facilitate OR teaching OR teach* OR train OR promote OR educate OR support OR guide OR coach OR instruct OR supervise) AND (“qualitative research” OR qualitative OR “qualitative study” OR phenomenology* OR phenomenograph* OR “grounded theory” OR qualitative* OR “content analysis” OR”thematic analysis” OR interview* OR “focus group*” OR interpretat* OR descriptive* OR narrative* OR ethnograph* OR “mixed method”) Filters: peer reviewed and publication year from 1970 onwards
ERIC	Search 27 March 2020
	(“physical therapist*” OR physiotherapist* OR “physical therap*” OR physiotherap* OR Undergraduate OR pre-registration OR post-registration OR postgraduate OR baccalaureate OR diploma OR “continuing education” OR pre- licensure OR post-licensure OR student OR teacher OR supervisor OR clinical instructor OR educator OR “higher education” OR “continuing education” OR “clinical setting” OR “clinical placement” OR “practice placement” OR internship OR practicum OR “healthcare facility”) AND (perceptions OR experiences OR conceptions OR attitudes OR feelings OR views OR beliefs) AND (“human movement” OR movement OR “movement quality” OR “human movement quality” OR “human movement embod*” OR “movement embod*” OR “movement phenomena” OR “human movement phenomena” OR “movement phenomenon” OR “human movement phenomenon” OR “movement perspective” OR “human movement perspective” OR “movement element” OR “human movement element” OR “movement process” OR “human movement process” OR “movement aspect” OR “human movement aspect” OR “movement communication” OR “human movement communication” OR “movement dialogue” OR “human movement dialogue” OR “movement description” OR “human movement description” OR “movement vocabulary” OR “human movement vocabulary” OR “movement awareness” OR “human movement awareness” OR “movement sensation” OR “human movement sensation” OR “movement control” OR “human movement control” OR “movement skill” OR “human movement skill” OR “movement knowledge” OR “human movement knowledge” OR “movement knowing” OR “human movement knowing” OR “human movement pedagog*” OR “movement pedagog*”) AND (learning OR learn* OR reflect OR assimilate OR adopt OR understand OR develop OR espouse OR enhance OR facilitate OR teaching OR teach* OR train OR promote OR educate OR support OR guide OR coach OR instruct OR supervise) AND (“qualitative research” OR qualitative OR “qualitative study” OR phenomenology* OR phenomenograph* OR “grounded theory” OR qualitative* OR “content analysis” OR “thematic analysis” OR interview* OR “focus group*” OR interpretat* OR descriptive* OR narrative* OR ethnograph* OR “mixed method”) Filters: peer reviewed and publication year from 1970 onwards
PsycINFO	Search 27 March 2020
	(“physical therapist*” OR physiotherapist* OR “physical therap*” OR physiotherap* OR Undergraduate OR pre-registration OR post-registration OR postgraduate OR baccalaureate OR diploma OR “continuing education” OR pre-licensure OR post-licensure OR student OR teacher OR supervisor OR clinical instructor OR educator OR “higher education” OR “continuing education” OR “clinical setting” OR “clinical placement” OR “practice placement” OR internship OR practicum OR “healthcare facility”) AND (perceptions OR experiences OR conceptions OR attitudes OR feelings OR views OR beliefs) AND (“human movement” OR movement OR “movement quality” OR “human movement quality” OR “human movement embod*” OR “movement embod*” OR “movement phenomena” OR “human movement phenomena” OR “movement phenomenon” OR “human movement phenomenon” OR “movement perspective” OR “human movement perspective” OR “movement element” OR “human movement element” OR “movement process” OR “human movement process” OR “movement aspect” OR “human movement aspect” OR “movement communication” OR “human movement communication” OR “movement dialogue” OR “human movement dialogue” OR “movement description” OR “human movement description” OR “movement vocabulary” OR “human movement vocabulary” OR “movement awareness” OR “human movement awareness” OR “movement sensation” OR “human movement sensation” OR “movement control” OR “human movement control“OR “movement skill” OR “human movement skill” OR “movement knowledge” OR “human movement knowledge” OR “movement knowing” OR “human movement knowing” OR “human movement pedagog*” OR “movement pedagog*”) AND (learning OR learn* OR reflect OR assimilate OR adopt OR understand OR develop OR espouse OR enhance OR facilitate OR teaching OR teach* OR train OR promote OR educate OR support OR guide OR coach OR instruct OR supervise) AND (“qualitative research” OR qualitative OR “qualitative study” OR phenomenology* OR phenomenograph* OR “grounded theory” OR qualitative* OR “content analysis” OR “thematic analysis” OR interview* OR “focus group*” OR interpretat* OR descriptive* OR narrative* OR ethnograph* OR “mixed method”) Filters: peer reviewed and publication year from 1970 onwards
FINNA	Search 9 April 2020
	(“physical therapist*” OR physiotherapist* OR “physical therap*” OR physiotherap* OR Undergraduate OR pre-registration OR post-registration OR postgraduate OR baccalaureate OR diploma OR “continuing education” OR pre-licensure OR post-licensure OR student OR teacher OR supervisor OR clinical instructor OR educator OR “higher education” OR “continuing education” OR “clinical setting” OR “clinical placement” OR “practice placement” OR internship OR practicum OR “healthcare facility”) AND (perceptions OR experiences OR conceptions OR attitudes OR feelings OR views OR beliefs) AND (“human movement” OR movement OR “movement quality” OR “human movement quality” OR “human movement embod*” OR “movement embod*” OR “movement phenomena” OR “human movement phenomena” OR “movement phenomenon” OR “human movement phenomenon” OR “movement perspective” OR “human movement perspective” OR “movement element” OR “human movement element” OR “movement process” OR “human movement process” OR “movement aspect” OR “human movement aspect” OR “movement communication” OR “human movement communication” OR “movement dialogue” OR “human movement dialogue” OR “movement description” OR “human movement description” OR “movement vocabulary” OR “human movement vocabulary” OR “movement awareness” OR “human movement awareness” OR “movement sensation” OR “human movement sensation” OR “movement control” OR “human movement control“OR “movement skill” OR “human movement skill” OR “movement knowledge” OR “human movement knowledge” OR “movement knowing” OR “human movement knowing” OR “human movement pedagog*” OR “movement pedagog*”) AND (learning OR learn* OR reflect OR assimilate OR adopt OR understand OR develop OR espouse OR enhance OR facilitate OR teaching OR teach* OR train OR promote OR educate OR support OR guide OR coach OR instruct OR supervise) AND (“qualitative research” OR qualitative OR “qualitative study” OR phenomenology* OR phenomenograph* OR “grounded theory” OR qualitative* OR “content analysis” OR “thematic analysis” OR interview* OR “focus group*” OR interpretat* OR descriptive* OR narrative* OR ethnograph* OR “mixed method”) Filter: publication year from 1970 onwards
MEDIC	Search 9 April 2020
	(“physical therapist*” OR physiotherapist* OR “physical therap*” OR physiotherap* OR Undergraduate OR pre-registration OR post-registration OR postgraduate OR baccalaureate OR diploma OR “continuing education” OR pre-licensure OR post-licensure OR student OR teacher OR supervisor OR clinical instructor OR educator OR “higher education” OR “continuing education” OR “clinical setting” OR “clinical placement” OR “practice placement” OR internship OR practicum OR “healthcare facility”) AND (perceptions OR experiences OR conceptions OR attitudes OR feelings OR views OR beliefs) AND (“human movement” OR movement OR “movement quality” OR “human movement quality” OR “human movement embod*” OR “movement embod*” OR “movement phenomena” OR “human movement phenomena” OR “movement phenomenon” OR “human movement phenomenon” OR “movement perspective” OR “human movement perspective” OR “movement element” OR “human movement element” OR “movement process” OR “human movement process” OR “movement aspect” OR “human movement aspect” OR “movement communication” OR “human movement communication” OR “movement dialogue” OR “human movement dialogue” OR “movement description” OR “human movement description” OR “movement vocabulary” OR “human movement vocabulary” OR “movement awareness” OR “human movement awareness” OR “movement sensation” OR “human movement sensation” OR “movement control” OR “human movement control“OR “movement skill” OR “human movement skill” OR “movement knowledge” OR “human movement knowledge” OR “movement knowing” OR “human movement knowing” OR “human movement pedagog*” OR “movement pedagog*”) AND (learning OR learn* OR reflect OR assimilate OR adopt OR understand OR develop OR espouse OR enhance OR facilitate OR teaching OR teach* OR train OR promote OR educate OR support OR guide OR coach OR instruct OR supervise) AND (“qualitative research” OR qualitative OR “qualitative study” OR phenomenology* OR phenomenograph* OR “grounded theory” OR qualitative* OR “content analysis” OR “thematic analysis” OR interview* OR “focus group*” OR interpretat* OR descriptive* OR narrative* OR ethnograph* OR “mixed method”) Filter: publication year from 1970 onwards

### Appendix 2. Characteristics of included studies

**Table:**

Study	Data collection methods and analysis methods	Country	Phenomena of interest	Setting/context/culture	Participant characteristics and sample size	Description of main results
Ahola et al. (2017)^1^	Phenomenographic approach, reflective diaries, semi-structured group interviews	Finland	Physiotherapy students’ conceptions of movement quality	Physiotherapy BSc Program, higher education	Physiotherapy students (*n*=6) two second-year, four third-year students (5 female, 1 male, aged 22 — 33)	Three descriptive categories: reflecting on and deepening understandings of movement quality
Covington and Barcinas (2017)^2^	Qualitative research, constructionist grounded theory, semi-structured interviews, and video-recording situational analysis	USA	Physical therapist clinical instructors’ facilitation of students’ emerging integration of movement in practice	Physical therapy students’ clinical practice internships	Physical therapist clinical instructors (*n*=5), 18.5 years of clinical instructor experience, physical therapist students (*n*=5)	Five thematic behaviours of a clinical instructor to facilitate students’ use of movement in practice
Denneny et al. (2020)^3^	Phenomenological approach, observations, enriched by participants’ accounts, theory-laden thematic analysis	UK	Psychologically informed physiotherapists’ behaviours and intentions to help people with chronic pain	Outpatient pain management, physiotherapy departments, teaching hospital	Experienced physiotherapists (*n*=4)	Findings demonstrated cognitive and behavioural therapy competences in the model (CBT practitioners)
Durham et al. (2009)^4^	Mixed-methodology approach; video- recordings, semi-structured interviews; quantitative, qualitative, and mixed methods	UK	Use of attentional focus feedback in retraining of hemiplegic arm	Stroke rehabilitation hospitals, inpatient and outpatient departments	Physiotherapists (*n*= 8), all female: one junior, six senior, one stroke clinical specialist. Patients (*n*=8): seven male, one female (mean age 56 years, mean time post stroke 7.1 months)	Physiotherapists used instructions and statements of motivation more than feedback and directed the patient’s attention more to body movement than movement effects
Embrey et al. (1996)^5^	Retrospective think-aloud procedures, semi-structured interviews, content analysis	USA	Insight into characteristics of clinical decision making in paediatric physical therapy	Outpatient centres (*n*=4) for children with cerebral palsy	Experienced (*n*=3), inexperienced (*n*=3) paediatric physical therapists, children (*n*=18) with cerebral palsy, aged 2 — 10	Four characteristics of clinical decision making in paediatric physical therapy
Embrey and Hylton (1996)^6^	Retrospective think-aloud procedures, transcripts based on video-recordings of therapy sessions, content analysis	USA	Clinical applications of movement scripts by experienced and novice physical therapists	Outpatient centres for children with cerebral palsy	Experienced (*n*=3) and novice (*n*=3) paediatric physical therapists, children (*n*=18) with cerebral palsy, aged 2 — 9	Experienced and novice therapists applied cognitive schemata called movement scripts
Farjoun et al. (2020)^7^	Phenomenological, non-structured interviews, thematic analysis	Spain	Essence of the Bobath concept in the physiotherapy of children of cerebral palsy	Spanish Association of Bobath-trained therapists with paediatric specialization	Spanish BC-trained paediatric physical therapists (*n*=10): 8 female (mean age 4.9), work experience 19.7 and experience using BC 14.9 years	Essential components for understanding and defining the Bobath concept as it is practised
Hedlund and Gyllensten (2013)^8^	Qualitative approach, interviews, content analysis	Sweden	Physiotherapists’ experience of BBAT with patients with schizophrenia disorders	Southern and central parts of Sweden, five different cities, Institute of BBAT in Sweden	Physiotherapists (*n*=8): all female, aged 30 — 67, work experience 6 — 31 years	Themes found: encountering, discovery of embodiment, inner space and outer world.
Johnson et al. (2013)^9^	Qualitative, semi-structured interviews, content analysis	UK	Internal and external focus of attention during gait re-education following stroke	Hospital, neurological rehabilitation setting	Physical therapists (*n*=8): all female, 3 — 12 years of experience in stroke rehabilitation, Patients (*n*=8): five male, three female, aged 36 — 85 years, 7 — 216 days post stroke	Physical therapists frequently encouraged patients to be aware of their movements and their performance, instructions and feedback used frequently
(Lindvall et al., 2016)^10^	Descriptive, semi-structured interviews, content analysis	Sweden	Experiences of BBAT from the perspective of both patients with stroke, and physiotherapists	Primary health care centre (*n*=4)	Patients with stroke (*n*=21): ten female, eleven male, aged 42 — 80, Physiotherapists (*n*=4): all female, clinical experience in BBAT 15 — 20 years	Findings showed integration movements in daily life and a feeling of harmony
Normann et al. (2014)^11^	Phenomenological, interviews, content analysis	Norway	Clinical guidance of physiotherapists treating people with MS	Regional hospital, outpatient clinics for people with MS	Community physiotherapists (*n*=9): seven female, two male, experience in treatment of MS	Physiotherapists identified how a familiar patient’s participation in authentic movement analysis was significant for professional development
Ryan et al. (2020)^12^	Descriptive interpretive approach, semi-structured interviews, content analysis	Canada	Physiotherapists’ use of motor learning strategies in gait-based interventions for children with CP	Rehabilitation hospital (*n*= 3), Lokomat clinical trials of children with CP	Physiotherapists (*n*=8): all female, working in one rehabilitation hospital of Lokomat clinical trials of children with CP	Findings highlighted the influence of the child, therapist, and treatment approach on the implementation of motor learning strategies in gait-based physiotherapy intervention for children with CP
Skjaerven et al. (2010)^13^	Phenomenological, semi-structured interviews, content analysis	Norway	Physical therapists’ promotion of movement quality in their usual clinical settings	Physical therapist experts from the fields of neurology, primary health care, and mental health	Physical therapists (*n*=15): thirteen female, two male, aged 30 — 68, work experience 10 — 39 years	Findings demonstrated specific attitudes and skills of physical therapist experts in promoting movement quality in their clinical practice
Skjaerven et al. (2008)^14^	Phenomenological, interviews, content analysis	Norway	Essential features and characteristics of movement quality reflecting a group of expert physiotherapists’ understanding of the phenomenon	Neurological physiotherapy, primary health care, and psychiatric/psychosomatic physiotherapy	Physiotherapists (*n*=15): thirteen female, two male, seven worked at university hospital, eight in primary health care, all recognized experts	Movement quality was seen as a unifying phenomenon, representing a synthesis of biomechanical, physiological, psycho-sociocultural, and existential processes
Stephenson and Stephens (2018)^15^	Phenomenological, semi-structured interviews, thematic analysis	UK	Physiotherapists’ experiences of robotic therapy in upper limb rehabilitation in a stroke rehabilitation centre	Stroke rehabilitation centre in the north of England	Physiotherapists (*n* = 6): three female, three male, working with robotics at a stroke rehabilitation centre	Five interdependent themes were identified, which focused on individualized care
Svensen and Bergland (2007)^16^	Qualitative, in-depth interviews, content analysis	Norway	Learning through bodily experience to facilitate the development of empathy in first-year physiotherapy students	Physiotherapy BSc Program, higher education	Physiotherapy students (*n*=9): six female, three men, aged 22 — 26	Developing empathy among first-year students involves developing their ability to recognize, to be involved, and to be open-minded
Wohlin Wottrich et al. (2004)^17^	Descriptive, comparative approach, observations, semi-structured interviews, content analysis	Sweden	Characteristics of physiotherapy sessions from perspectives of patients with stroke and therapists	Rehabilitation units (neurological, geriatric) in the Stockholm area	Physiotherapists (*n*=10): aged 25 — 60, Patients (*n*=9): aged 45 — 88, post stroke .5 — 48 months	Findings showed differences between physiotherapists’ and patients’ descriptions of physiotherapy sessions’ characteristics
